# Bulbar involvement and cognitive features in amyotrophic lateral sclerosis: a retrospective study on 347 patients

**DOI:** 10.3389/fnagi.2023.1217080

**Published:** 2023-07-20

**Authors:** Edoardo Nicolò Aiello, Federica Solca, Silvia Torre, Valerio Patisso, Alberto De Lorenzo, Mauro Treddenti, Eleonora Colombo, Alessio Maranzano, Claudia Morelli, Alberto Doretti, Federico Verde, Vincenzo Silani, Nicola Ticozzi, Barbara Poletti

**Affiliations:** ^1^Department of Neurology and Laboratory of Neuroscience, IRCCS Istituto Auxologico Italiano, Milan, Italy; ^2^Neurology Residency Program, Università Degli Studi di Milano, Milan, Italy; ^3^Department of Pathophysiology and Transplantation, “Dino Ferrari” Center, Università Degli Studi di Milano, Milan, Italy; ^4^Department of Oncology and Hemato-Oncology, Università degli Studi di Milano, Milan, Italy

**Keywords:** bulbar, Frontotemporal Degeneration, cognition, neuropsychology, amyotrophic lateral sclerosis

## Abstract

**Background:**

This study aimed at clarifying the role of bulbar involvement (BI) as a risk factor for cognitive impairment (CI) in non-demented amyotrophic lateral sclerosis (ALS) patients.

**Methods:**

Data on *N* = 347 patients were retrospectively collected. Cognition was assessed via the Edinburgh Cognitive and Behavioral ALS Screen (ECAS). On the basis of clinical records and ALS Functional Rating Scale-Revised (ALSFRS-R) scores, BI was characterized as follows: (1) BI at onset—from medical history; (2) BI at testing (an ALSFRS-R-Bulbar score ≤11); (3) dysarthria (a score ≤3 on item 1 of the ALSFRS-R); (4) severity of BI (the total score on the ALSFRS-R-Bulbar); and (5) progression rate of BI (computed as 12-ALSFRS-R-Bulbar/disease duration in months). Logistic regressions were run to predict a below- vs. above-cutoff performance on each ECAS measure based on BI-related features while accounting for sex, disease duration, severity and progression rate of respiratory and spinal involvement and ECAS response modality.

**Results:**

No predictors yielded significance either on the ECAS-Total and -ALS-non-specific or on ECAS-Language/-Fluency or -Visuospatial subscales. BI at testing predicted a higher probability of an abnormal performance on the ECAS-ALS-specific (*p* = 0.035) and ECAS-Executive Functioning (*p* = 0.018). Lower ALSFRS-R-Bulbar scores were associated with a defective performance on the ECAS-Memory (*p* = 0.025). No other BI-related features affected other ECAS performances.

**Discussion:**

In ALS, the occurrence of BI itself, while neither its specific features nor its presence at onset, might selectively represent a risk factor for executive impairment, whilst its severity might be associated with memory deficits.

## 1. Background

Bulbar involvement (BI) has been historically linked to cognitive impairment (CI) in amyotrophic lateral sclerosis (ALS) (Zago et al., 2022). However, bulbar onset and dysarthria have been only recently acknowledged, at a meta-analytic level, as actual risk factors for frontotemporal-*spectrum* disorders in this population (Yang et al., 2021). Relatedly, recent neuropathological evidence has supported such a phenotypic association within a network-based framework, according to which medullary pathology would readily spread to bulbar sensory-motor cortices and, in turn, to the frontal and temporal areas connected to them (Shellikeri et al., 2020).

However, among the studies included in the abovementioned meta-analysis by Yang et al. (2021) for the specific aim of testing whether bulbar onset and dysarthria could represent risk factors for CI in ALS, only a minority (i.e., 3 out of 14) performed some sort of adjustment for bulbar confounders during task execution (Kasper et al., 2016; Trojsi et al., 2017; Watanabe et al., 2020). Moreover, none of these reports employed an ALS-specific measure that could control for BI as much as possible—such as the Edinburgh Cognitive and Behavioral ALS Screen (ECAS) (Abrahams et al., 2014). At most, among such studies (Yang et al., 2021), the confounding effect of BI was accounted for via mere attempts, performed either *a priori*—e.g., by selecting untimed cognitive tests that minimally relied on verbal responses (Massman et al., 1996) or by excluding patients unable to execute a minimum number of cognitive tests (Oh et al., 2014)—or *a posteriori*—e.g., by analytically comparing the rates of CI between dysarthric and non-dysarthric patients (Massman et al., 1996; Rippon et al., 2006). Hence, given that accommodating for BI when assessing cognition in ALS patients is essential (Woolley and Rush, 2017), the conclusions drawn by Yang et al. (2021) appear to be based, to a non-negligible extent, on biased studies.

Unfortunately, the same methodological issues also affect other relevant reports on the topic that were not included in Yang et al. (2021) meta-analysis (Portet et al., 2001; Schreiber et al., 2005; Röttig et al., 2006; Sterling et al., 2010; Morimoto et al., 2012; Zalonis et al., 2012; Mannarelli et al., 2014; Burke et al., 2016). Therein, the attempts to control for the confounding effect of BI on cognition in ALS, which were mostly performed *a posteriori*, led to indeterminate conclusions (Portet et al., 2001; Schreiber et al., 2005; Röttig et al., 2006; Sterling et al., 2010; Morimoto et al., 2012; Zalonis et al., 2012; Mannarelli et al., 2014; Burke et al., 2016). Most importantly, the vast majority of these works (Massman et al., 1996; Portet et al., 2001; Schreiber et al., 2005; Rippon et al., 2006; Röttig et al., 2006; Sterling et al., 2010; Morimoto et al., 2012; Zalonis et al., 2012; Mannarelli et al., 2014; Oh et al., 2014; Burke et al., 2016; Woolley and Rush, 2017; Yang et al., 2021) approached the association between BI and CI in ALS via simple correlational/predictive statistics. Indeed, most of these studies (1) did not disentangle the contribution of BI at onset from that at the time of cognitive testing, (2) did not focus on the severity and progression rate of BI itself, and (3) did not covary for other motor-functional features that possibly increase the risk of CI this population—i.e., respiratory dysfunctions (Huynh et al., 2020b; Shah et al., 2021) and an advanced disease (Crockford et al., 2018; Chiò et al., 2019).

Hence, by addressing a large cohort of non-demented ALS patients, the present study aimed at assessing, via multiple regression models, the association between a comprehensive set of BI-related features and a disease-specific cognitive measure (i.e., the ECAS) net of other motor-functional variables.

## 2. Methods

### 2.1. Participants

The current retrospective cohort included *N* = 347 ALS patients (Brooks et al., 2000) consecutively referred to IRCCS Istituto Auxologico Italiano, Milano, Italy between 2016 and 2023 who were administered the ECAS (Poletti et al., 2016) and for whom onset data and ALS Functional Rating Scale-Revised (ALSFRS-R) (Cedarbaum et al., 1999) scores were available. Patients did not present with (1) a co-morbid diagnosis of frontotemporal dementia (FTD) (Gorno-Tempini et al., 2011; Rascovsky et al., 2011), (2) ALS-unrelated neurological/psychiatric disorders, (3) severe/unstable general-medical conditions, and (4) uncorrected sensory deficits.

### 2.2. Materials

The cognitive section of the Italian ECAS (Poletti et al., 2016) (*range* = 0–136) includes 5 subscales assessing *Language* (ECAS-L; *range* = 0–28), *Fluency* (ECAS-F; *range* = 0–24), *Executive Functioning* (ECAS-EF; *range* = 0–48), *Memory* (ECAS-M; *range* = 0–24), and *Visuospatial* abilities (ECAS-VS; *range* = 0–12). ECAS-ALS-specific (i.e., ECAS-L + ECAS-F + ECAS-EF; *range* = 0–100) and -non-specific subscores (i.e., ECAS-M + ECAS-VS; *range* = 0–36) were also computed. ALSFRS-R items (Cedarbaum et al., 1999) were grouped as follows: (1) ALSFRS-R-Bulbar (items 1–3; *range* = 0–12); (2) ALSFRS-R-Spinal (items 4–9; *range* = 0–24); and (3) ALSFRS-R-Respiratory (items 10–12; *range* = 0–12). Progression rate (ΔFS) was computed according to Kimura et al. (2006) formula for each ALSFRS-R subscale—i.e., by weighting on disease duration (in months) the difference between the maximum and the actual ALSFRS-R subscore. Disease staging was retrieved according to both King’s college (Roche et al., 2012) and Milano-Torino (MiToS) (Chiò et al., 2015) systems.

### 2.3. Statistics

Logistic regressions were run separately for each ECAS measure by addressing, as the outcome, a below- vs. above-cutoff performance [based on age- and education-stratified Italian normality thresholds (Poletti et al., 2016)], and, as predictors, (1) bulbar onset—retrieved from patients’ medical history—(2) presence of BI at testing—defined as an ALSFRS-R-Bulbar score ≤11—(3) presence of dysarthria—defined as a score ≤3 on item 1 of the ALSFRS-R—(4) severity of BI—i.e., the total score on the ALSFRS-R-Bulbar—and (5) progression rate of BI—i.e., ΔFS-Bulbar scores. Within these models, sex, disease duration (in months), severity and progression rate of respiratory and spinal involvement (i.e., ALSFRS-R-Spinal, ALSFRS-R-Respiratory, ΔFS-Spinal and ΔFS-Respiratory scores) and ECAS response modality (i.e., oral vs. written) were covaried. Collinearity was diagnosed in the presence of a Variance Inflation Factor (VIF) >10 and of a Tolerance Index (TI) < 0.1.

Analyses were run via IBM^®^ SPSS^®^ Statistic (IBM Corp., 2021) and jamovi 2.3 (the jamovi project, 2022).

## 3. Results

Table 1 summarizes patients’ background and clinical features, and Table 2 reports the complete results of the logistic regression models.

**TABLE 1:** Patients’ demographic, clinical, and cognitive measures.

*N*	347
Sex (male/female)	218/129
Age (years)	63.2 ± 11.5 (20–88)
Education (years)	11.6 ± 4.3 (5–24)
Disease duration (months)	18.7 ± 21.2 (1–264)
**ALSFRS-R**	
Total	38.2 ± 6.7 (12–48)
Bulbar	10.2 ± 2.3 (1–12)
Spinal	16.8 ± 5.8 (0–24)
Respiratory	11.2 ± 1.6 (0–12)
**Δ FS**	
Total	0.8 ± 0.9 (0–6.3)
Bulbar	0.2 ± 0.3 (0–2.8)
Spinal	0.6 ± 0.7 (0–4.5)
Respiratory	0.1 ± 0.2 (0–1.3)
NIV (%)	4.9%
PEG (%)	0.3%
**Bulbar involvement (%)**	
At onset	24.8%
At testing	55.3%
Dysarthria (%)	45.8%
**Genetics (*N*)**	
*C9orf72*	22
*TARDBP*	12
*SOD1*	9
**King’s (%)**	
Stage 1	36.6%
Stage 2	32.3%
Stage 3	25.9%
Stage 4	5.2%
**MiToS (%)**	
Stage 0	66.6%
Stage 1	21.6%
Stage 2	10.7%
Stage 3	1.2
**ECAS**	
Total	99.5 ± 19.3 (31–129)
Impaired (%)	33.4%
ALS-specific	73.4 ± 15.5 (21–97)
Impaired (%)	32.0%
ALS-non-specific	26.1 ± 5.2 (9–34)
Impaired (%)	23.3%
Language	23.5 ± 4 (9–28)
Impaired (%)	22.5%
Fluency	16.3 ± 5.7 (0–24)
Impaired (%)	21.3%
Executive functioning	33.7 ± 8.1 (7–48)
Impaired (%)	22.2%
Memory	14.7 ± 4.7 (1–22)
Impaired (%)	21.0%
Visuo-spatial	11.3 ± 1.1 (5–12)
Impaired (%)	8.6%

ALS, amyotrophic laterals sclerosis; ALSFRS-R, amyotrophic lateral sclerosis functional rating scale-revised; ΔFS, progression rate; ECAS, Edinburgh cognitive and behavioral ALS screen; MiToS, Milano-Torino staging; NIV, non-invasive ventilation; PEG, percutaneous endoscopic gastrostomy.

**TABLE 2:** Effects of bulbar features on ECAS performances as yielded by the logistic regression models.

Outcome	Predictor	*b*	OR [CI 95%]	*z*	*p*
**ECAS-Total**					
**(Impaired vs. unimpaired)**					
	ALSFRS-R-bulbar	−0.03	0.97 [0.77, 1.22]	−0.28	0.780
	ΔFS-bulbar	−0.24	0.79 [0.16, 3.44]	−0.32	0.752
	BI at onset (present vs. absent)	0.39	1.47 [0.65, 3.32]	0.94	0.347
	BI at testing (present vs. absent)	0.81	2.24 [0.94, 5.29]	1.84	0.065
	Dysarthria (present vs. absent)	−0.51	0.60 [0.24, 1.51]	−1.08	0.279
**ECAS-ALS-specific**					
**(Impaired vs. unimpaired)**					
	ALSFRS-R-bulbar	−0.09	0.92 [0.73, 1.15]	−0.74	0.459
	ΔFS-bulbar	0.00	1.00 [0.22, 4.32]	0.00	0.999
	BI at onset (present vs. absent)	0.35	1.42 [0.63, 3.21]	0.85	0.398
	BI at testing (present vs. absent)	0.92	2.50 [1.06, 5.90]	2.11	0.035*
	Dysarthria (present vs. absent)	−0.80	0.45 [0.18, 1.12]	−1.71	0.087
**ECAS-ALS-non-specific**					
**(Impaired vs. unimpaired)**					
	ALSFRS-R-bulbar	−0.21	0.82 [0.64, 1.03]	−1.69	0.092
	ΔFS-bulbar	−0.88	0.42 [0.07, 2.06]	−1.04	0.298
	BI at onset (present vs. absent)	−0.64	0.53 [0.21, 1.28]	−1.40	0.163
	BI at testing (present vs. absent)	−0.72	0.49 [0.13, 1.45]	−1.20	0.231
	Dysarthria (present vs. absent)	0.92	2.50 [0.80, 9.64]	1.47	0.141
**ECAS-Language**					
**(Impaired vs. unimpaired)**					
	ALSFRS-R-bulbar	−0.03	0.97 [0.76, 1.26]	−0.20	0.838
	ΔFS-bulbar	0.84	2.33 [0.44, 11.78]	1.02	0.306
	BI at onset (present vs. absent)	0.05	1.06 [0.42, 2.59]	0.12	0.907
	BI at testing (present vs. absent)	0.76	2.15 [0.86, 5.25]	1.66	0.097
	Dysarthria (present vs. absent)	−0.84	0.43 [0.16, 1.7]	−1.67	0.095
**ECAS-Fluency**					
**(Impaired vs. unimpaired)**					
	ALSFRS-R-bulbar	−0.21	0.81 [0.61, 1.07]	−1.48	0.138
	ΔFS-bulbar	−1.16	0.31 [0.02, 2.55]	−0.97	0.330
	BI at onset (present vs. absent)	−0.09	0.91 [0.34, 2.37]	−0.19	0.851
	BI at testing (present vs. absent)	0.64	1.89 [0.70, 4.91]	1.30	0.195
	Dysarthria (present vs. absent)	−0.71	0.49 [0.18, 1.43]	−1.32	0.186
**ECAS-Executive Functioning**					
**(Impaired vs. unimpaired)**					
	ALSFRS-R-bulbar	−0.11	0.90 [0.71, 1.14]	−0.88	0.377
	ΔFS-bulbar	0.87	2.39 [0.50, 11.17]	1.12	0.265
	BI at onset (present vs. absent)	0.20	1.22 [0.50, 2.97]	0.44	0.658
	BI at testing (present vs. absent)	1.11	3.03 [1.20, 7.57]	2.37	0.018*
	Dysarthria (present vs. absent)	−0.86	0.42 [0.16, 1.11]	−1.76	0.078
**ECAS-Memory**					
**(Impaired vs. unimpaired)**					
	ALSFRS-R-bulbar	−0.28	0.75 [0.59, 0.96]	−2.24	0.025*
	ΔFS-bulbar	−0.44	0.64 [0.11, 3.31]	−0.51	0.607
	BI at onset (present vs. absent)	−0.63	0.53 [0.20, 1.34]	−1.32	0.187
	BI at testing (present vs. absent)	−0.52	0.59 [0.18, 1.68]	−0.93	0.354
	Dysarthria (present vs. absent)	0.25	1.28 [0.42, 4.48]	0.42	0.673
**ECAS-Visuospatial**					
**(Impaired vs. unimpaired)**					
	ALSFRS-R-bulbar	−0.18	0.84 [0.59, 1.16]	−1.06	0.291
	ΔFS-bulbar	−0.21	0.81 [04, 9.48]	−0.15	0.877
	BI at onset (present vs. absent)	0.05	1.06 [0.27, 3.98]	0.08	0.937
	BI at testing (present vs. absent)	−0.39	0.68 [0.03, 4.30]	−0.35	0.729
	Dysarthria (present vs. absent)	0.79	2.20 [0.33, 44.02]	0.70	0.486

ALSFRS-R, amyotrophic lateral sclerosis functional rating scale-revised; BI, bulbar involvements; ΔFS, progression rate; ECAS, Edinburgh cognitive and behavioral ALS screen. Model coefficients are computed net of sex, disease duration (in months), ALSFRS-R-spinal and -respiratory scores, ΔFS-spinal and -respiratory scores and response modality (i.e., oral vs. written). *Significant statistic.

No collinearity was detected among predictors (VIF ≤5.41; TI ≥ 0.19). No target predictors yielded significance on the ECAS-Total (*p*s ≥ 0.065), ECAS-ALS-non-specific (*p*s ≥ 0.092), ECAS-L (*p*s ≥ 0.095), ECAS-F (*p*s ≥ 0.138), and ECAS-VS (*p*s ≥ 0.291). At variance, BI at testing was associated with a higher probability of an impaired performance on the ECAS-ALS-Specific (*b* = 0.92; *z* = 2.11; OR = 2.5, CI 95% [1.06, 5.9]). Indeed, patients with BI at testing were more likely to perform defectively on this subscale (*M* = 0.39; *SE* = 0.08) when compared to those without (*M* = 0.20; *SE* = 0.06). Such a finding happened to be carried by the ECAS-EF: indeed, the probability of an abnormal performance on the ECAS-EF was found to be significantly higher in patients with BI (*M* = 0.26; *SE* = 0.07) when compared to those without BI (*M* = 0.10; *SE* = 0.04) at testing (*b* = 1.11; *z* = 2.37; OR = 3.03, CI 95% [1.2, 7.57]). Finally, patients with lower ALSFRS-R-Bulbar scores were found to be more likely to perform defectively on the ECAS-M (*b* = −0.28; *z* = −2.24; OR = 0.75, CI 95% [0.59, 0.96]). Indeed, the probability of an abnormal performance on the ECAS-M was lower (*M* = 0.08; *SE* = 0.04) in patients with higher (*M* + 1*SD) ALSFRS-R-Bulbar scores, and higher (*M* = 0.24; *SE* = 0.06) in patients with lower (*M*-1*SD) scores on this measure. No other BI-related variables yielded significance (*p*s > 0.05).

## 4. Discussion

The present report clarifies the role of BI as a risk factor for CI in ALS by simultaneously encompassing, within a large patient cohort, an extensive range of both BI-related predictors and other motor-functional covariates, as well as by addressing a disease-specific measures of cognition that compensates for motor disabilities (i.e., the ECAS).

This study suggests that, net of overall motor-functional status, BI itself, and neither its presence at onset, severity, progression rate or phenotype (i.e., the occurrence of dysarthria), increases the probability of executive deficits non-demented ALS patients. Indeed, ALS patients with BI at testing were more likely to perform defectively on the ECAS-EF than those without BI at testing. Additionally, the current investigation suggests that patients with a more severe BI (i.e., lower ALSFRS-R-Bulbar scores) are more likely to present with memory deficits (i.e., an impaired performance on the ECAS-M).

The fact that neither BI at onset nor dysarthria herewith represented risk factors for CI in this ALS cohort is in contrast with Yang et al. (2021) meta-analysis: however the statistical approach chosen for this study, as well as the extensive range of BI-related features and motor-functional confounders taken into account, grant a larger extent of generalizability to the present results. At the same time, in respect to BI at onset, this report aligns with two previous meta-analyses on the cognitive phenotype of ALS (Raaphorst et al., 2010; Beeldman et al., 2016)—wherein no association was detected between BI at onset and an increased risk for CI. Relatedly, a report by Zalonis et al. (2012), which selectively aimed to test whether executive measures could discriminate bulbar- from spinal-onset ALS patients, failed to corroborate this hypothesis. As to the present lack of association between dysarthria and an increased risk for CI, such a finding is likely due to the fact that the cognitive measure herewith employed—i.e., the ECAS—aprioristically accommodates for this motor confounder.

Overall, the present report supports the view that, from a network-based perspective (Shellikeri et al., 2017, 2020), ALS patients with BI may present with a greater involvement of extra-motor cortices when compared to those without BI (Steinbach et al., 2021). This stance is also supported by the neuroradiological report by Cistaro et al. (2012), who showed that, when compared to patients without BI, bulbar ALS patients show distinct functional brain features that correlate with the degree of CI.

Remarkably, BI at testing herewith represented a risk factor only for executive dysfunction, as it did not affect either overall cognitive efficiency or other cognitive domains/functions. While this finding is consistent with previous reports on the topic, which commonly link BI to executive deficits in ALS (Schreiber et al., 2005; Sterling et al., 2010; Morimoto et al., 2012; Mannarelli et al., 2014; Burke et al., 2016), it does not align with the literature concerning the association between BI and language impairment in this population (Pinto-Grau et al., 2018; Aiello et al., 2022a,b; Sbrollini et al., 2022). This inconsistency might be due to a measure-related issue—since, as previously suggested (Aiello et al., 2022c; McMillan et al., 2022; Solca et al., 2023), the ECAS-L does not represent a comprehensive language measure in ALS. Thus, it is advised that future studies address the link between BI and language impairment in this population by employing an extensive set of language tests. Similarly, no bulbar feature was herewith found to be associated with verbal fluency deficits, despite this link being frequently reported in previous investigations (Kew et al., 1993; Abrahams et al., 1996, 1997, 2000, 2005). This result is surprising—given that phonemic fluency tasks included within the ECAS-F have systemically proven sensitive to executive dysfunctions in this population (Kew et al., 1993; Abrahams et al., 1996, 1997, 2000, 2005). At the same time, it might be hypothesized that, since these tasks aprioristically accommodate for bulbar confounders (Abrahams et al., 2014; Canu et al., 2023), the further, *a posteriori* analytical expedient aimed at controlling for other BI-related features might have resulted in the lack of detection of the abovementioned association. However, an association between the severity of BI and memory deficits has herewith yielded. Such a finding—which is consistent with previous reports (Schreiber et al., 2005)—might be linked to the one regarding the ECAS-EF, and thus accounted for by the fact that memory deficits in this population are, at least to an extent, secondary to executive dysfunctions (Consonni et al., 2017; Barulli et al., 2019).

This study have several limitations. First, the evaluation of bulbar features herewith relied either on patients’ medical history or on the dedicated ALSFRS-R subscale. Hence, future studies are necessary that employ specific clinical scales aimed at assessing BI in ALS (Yunusova et al., 2019)—e.g., the Center for Neurologic Study Bulbar Function Scale (CNS-BFS) (Smith et al., 2018). Relatedly, the present study relied solely on clinical measures, but not on instrumental examinations, which would have better characterized the nature and extent of BI of the present cohort (Yunusova et al., 2019). Third, it must be borne in mind that the ECAS subscale yields a first-level measure of cognition within each target domain or function. Hence, further investigations that embrace the present experimental design by addressing a battery of second-level, domain-/function-specific cognitive tests are needed. Fourth, this report solely addressed patients without a co-morbid diagnosis of FTD, thus not being informative of the role of BI as a risk factor for full-blown dementing states in this population. However, in this respect, it is worth noting that a recent report by the present research group (Colombo et al., 2023) suggested that BI, along with genetic risk factors (i.e., *C9orf72* hexanucleotide repeat expansion), are associated with concurrent behavioral variant-FTD. Finally, the retrospective nature of the current study does not allow to draw inferences on how the association between BI and CI in this population might change over time. Future studies that delve into such matter should be undertaken that involve a longitudinal design (Colombo et al., 2023) addressing technology-aided cognitive assessment procedures fully overcoming patients’ motor disabilities across all disease stages (Cipresso et al., 2011; Poletti et al., 2017a,b, 2018a,b).

In conclusions, this study suggests that the occurrence of BI itself, whilst neither its specific clinical characteristics nor its presence at disease onset, selectively represents a risk factor for executive impairment in non-demented ALS patients, as well as that the severity of BI might be associated with memory deficits in this population. Overall, the present findings suggest that non-demented ALS patients presenting with BI should be carefully assessed for their executive—and possibly mnestic—status, given that deficits within such a set of cognitive functions are known to detrimentally impact on patients’ prognosis (Poletti et al., 2018a; Huynh et al., 2020a).

## Data availability statement

This set of raw data is accessible under request because it includes sensitive information. Raw data have been stored at the following link: https://doi.org/10.5281/zenodo.8103679.

## Ethics statement

The studies involving human participants were reviewed and approved by the Ethics Committees of IRCCS Istituto Auxologico Italiano (I.D.: 2013_06_25). The patients/participants provided their written informed consent to participate in this study.

## Author contributions

EA: conceptualization, analyses, drafting, and revision. FS, ST, VP, ADL, MT, EC, and AM: data collection and revision. CM, AD, FV, and VS: resources and revision. BP and NT: resources, drafting, and revision. All authors contributed to the article and approved the submitted version.
